# Concordant and Discordant Regulation of Target Genes by miR-31 and Its Isoforms

**DOI:** 10.1371/journal.pone.0058169

**Published:** 2013-03-05

**Authors:** Yu-Tzu Chan, You-Chin Lin, Ruey-Jen Lin, Huan-Hsien Kuo, Wai-Cheng Thang, Kuo-Ping Chiu, Alice L. Yu

**Affiliations:** 1 Institute of Biochemistry and Molecular Biology, National Yang-Ming University, Taipei, Taiwan; 2 Genomics Research Center, Academia Sinica, Taipei, Taiwan; 3 Department of Pediatrics/Hematology-Oncology, University of California San Diego Medical Center, San Diego, California, United States of America; The Ohio State University, United States of America

## Abstract

It has been shown that imprecise cleavage of a primary or precursor RNA by Drosha or Dicer, respectively, may yield a group of microRNA (miRNA) variants designated as “isomiR”. Variations in the relative abundance of isoforms for a given miRNA among different species and different cell types beg the question whether these isomiRs might regulate target genes differentially. We compared the capacity of three miR-31 isoforms (miR-31-H, miR-31-P, and miR-31-M), which differ only slightly in their 5′- and/or 3′-end sequences, to regulate several known targets and a predicted target, Dicer. Notably, we found isomiR-31s displayed concordant and discordant regulation of 6 known target genes. Furthermore, we validated a predicted target gene, Dicer, to be a novel target of miR-31 but only miR-31-P could directly repress Dicer expression in both MCF-7 breast cancer cells and A549 lung cancer cells, resulting in their enhanced sensitivity to cisplatin, a known attribute of Dicer knockdown. This was further supported by reporter assay using full length 3′-untranslated region (UTR) of Dicer. Our findings not only revealed Dicer to be a direct target of miR-31, but also demonstrated that isomiRs displayed similar and disparate regulation of target genes in cell-based systems. Coupled with the variations in the distribution of isomiRs among different cells or conditions, our findings support the possibility of fine-tuning gene expression by miRNAs.

## Introduction

MicroRNAs (miRNAs) are a group of small non-coding RNAs containing ∼22 nt which are involved in many biological processes of normal and malignant cells [1]–[4]. During the traditional biogenesis of miRNA, the primary miRNA (pri-miRNA) is processed by Drosha and its cofactor Pasha to a ∼70 nt stem-loop-like precursor miRNA (pre-miRNA) in the nucleus. Upon exporting to the cytoplasm by Exportin 5, pre-miRNA is further trimmed by Dicer to the mature miRNA in double strand form. After unwinding of mature miRNA duplex, the guide strand is loaded into the RNA-induced silencing complex (RISC) through complementary pairing with the target site on the 3′-untranslated region (UTR) of target mRNAs to trigger either translational repression or mRNA degradation in mammalian system [5]–[7]. Several lines of evidence have shown that the expression of key proteins, including Drosha or Dicer, correlated with tumorigenesis and prognosis in a variety of cancers [8]–[12]. Although Dicer plays an important role in miRNA maturation and is implicated in several biological processes [13]–[15], the regulation of Dicer has proved to be complex. It has been shown that Dicer was regulated by miRNA *let-7* and miR-103/107 family [16], which constitutes a negative feedback loop [17], [18].

So far, over 1,500 human miRNAs have been identified and annotated in the miRBase (version 18.0) [19]. The use of large-scale deep sequencing technique further uncovered a group of miRNAs, which diverge from their miRBase annotated sequence at 5′- and/or 3′-ends, in both animals and plants [20]–[27]. Theoretically, isoforms of a specific miRNA could be generated by imprecise Drosha/Dicer cleavage of a pri-miRNA/pre-miRNA, leading to miRNAs sequences which match precisely to genomic sequence. Alternatively, isomiRs could be produced by enzymatic RNA editing or nucleotide extensions, yielding miRNAs with sequences matched to genome at every nucleotide except 3′-end. All of these miRNA variants are referred to as “isomiR" [23], [28], [29]. A review of literature and data mining of the reported sequencing studies have revealed that: (1) The most abundant isoform of miRNAs may differ from the current miRBase annotated sequence. For example, the major form of miR-142-5p in Argonaute (AGO)-IP product from Jurkat cells contains two additional C at the 5′-end, but lacks U at the 3′-end as compared to the miRBase annotated sequence [22], [30] (Figure S1A). (2) The expression pattern of isomiRs across *Drosophila melanogaster* development and tissues varies significantly [31]. (3) Even within the same cells, such as human umbilical vein endothelial cells (HUVEC), the most prevalent isoform of miRNAs may differ under normal and hypoxia stress (e.g. miR-30b-5p and miR-455-3p in Figure S1B) [32]. Such observation implies that the population of isomiRs may vary in different types of tissues/cells or environmental conditions and the submitted sequences in the miRBase may not be representative for all tissues and cells in a given species. Moreover, the 5′-end variations may result in isomiRs of the same miRNA bearing different seed sequence (2^nd^ to 8^th^ nt), which is the key target recognition element, leading to their differential regulation of target mRNAs. However, very few studies have tackled the issue whether these isomiRs with variations at 5′- and/or 3′-ends display identical functions. Using an acellular in vitro target RNA cleavage assay, Azuma-Mukai *et al.* demonstrated a difference in target cleavage ability between miR-142-5p and its variant which contained two extra nucleotides at the 5′-end [22]. In another study, cells were transfected with biotinylated miR-10a, miR-10b and their isomiRs to pull down bound mRNAs. Microarray analysis revealed that among hundreds of mRNA enriched in the miRNA pull-down, most mRNAs were common to their isomiR pull-downs, but some were unique to the specific isomiRs [33]. Thus, it’s possible that isomiRs may share certain common mRNA targets but not all mRNA targets. In this study, we investigated miR-31 isoforms to further address the issue of their target specificity and the biological functions at the cellular level.

## Results

### Variations in the Preponderance of miR-31 Isoforms in Different Type of Cells

**Figure 1:**
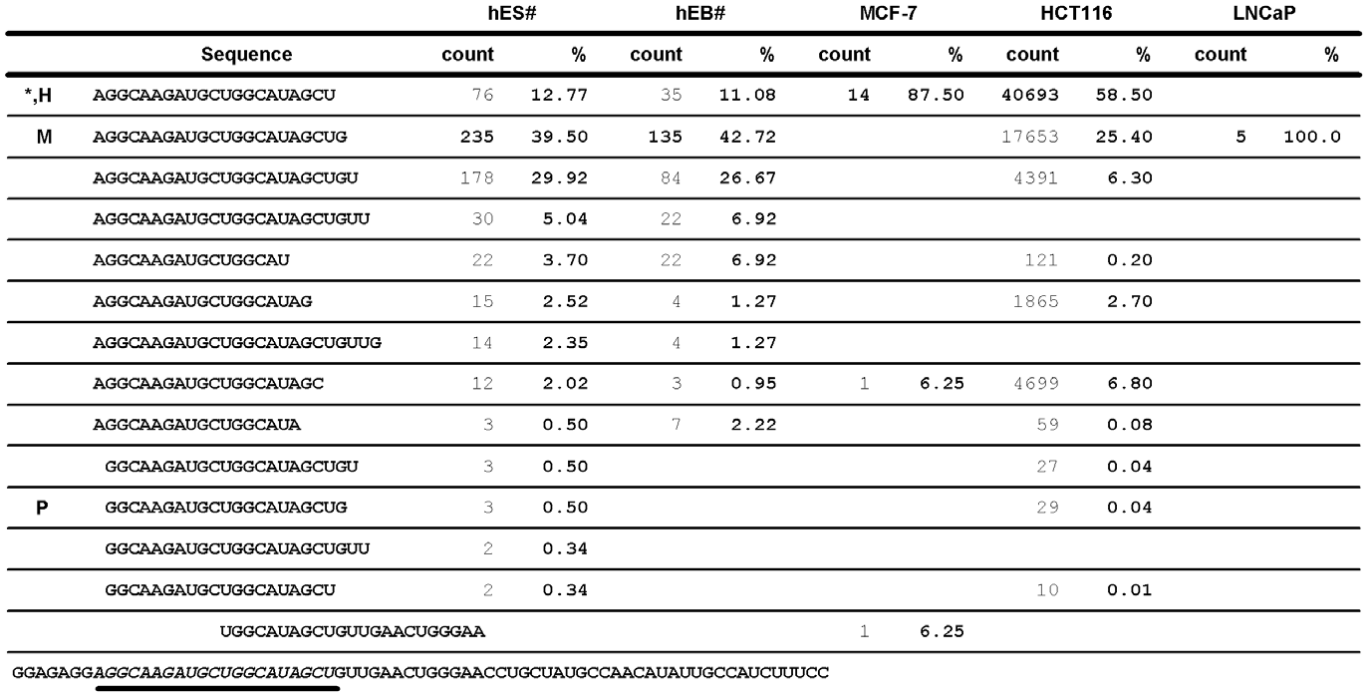
The most abundant isoform and the composition of miR-31 populations vary among five human cells. IsomiR-31s in MCF-7, HCT116, and LNCaP cells was analyzed by deep sequencing and compared to the reported miR-31 isoforms in human embryonic stem cell (hES)/embryonic body (hEB) culled from the supplementary data of Morin *et.al.* [23], [32]. The miR-31 precursor sequence is shown at the bottom. The sequences, which is underlined with thick line or marked with ^*^, is the current annotated miR-31 of human in miRBase (version 18.0). The occurrence of each sequence read is represented as the count shown in number. The percentage of each sequence indicates its occurrence in the whole population of miR-31 isoforms. In the miR-31 profile of HCT116 cells, most of sequences with counts of less than 10 were omitted from this figure. ^#^, the data were culled from the report of Morin *et al*. H, hsa-miR-31; the miR-31-H form. M, mmu-miR-31; the miR-31-M form. P, ptr-miR-31; the miR-31-P form.

**Figure 2:**
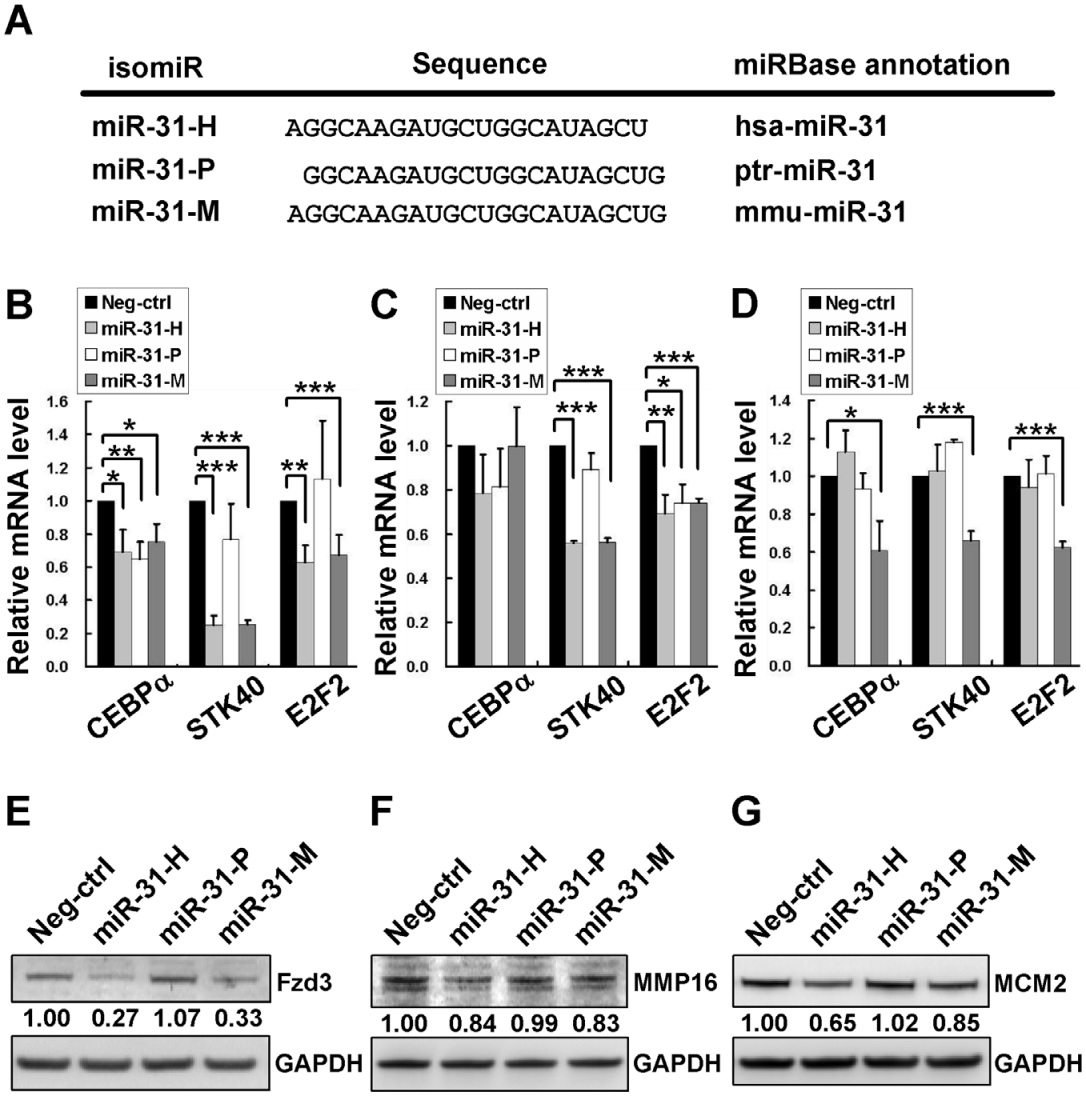
Concordant and discordant regulation of known target genes by isomiR-31s. The sequences of isomiRs of miR-31. MiR-31-H, miR-31-P, and miR-31-M represent hsa-miR-31, ptr-miR-31, and mmu-miR-31 in miRBase, respectively (A). CEBPα, STK40, and E2F2 mRNA expression in MDA-MB-231 cells (B), MCF-7 cells (C), and in HCT116 cells (D) were detected by RT-qPCR after transfection with synthetic oligos of isomiR-31s. The mRNA level of each gene was normalized to GAPDH mRNA. The normalized mRNA level of Neg-ctrl transfectant was set as 1.0 and then those of other isomiR-31 transfections were relative to it. The proteins levels of Fzd3 (E), MMP16 (F), and MCM2 (G) were determined in MDA-MB-231 cells transfected with 100 nM synthetic oligos by immunoblotting. GAPDH protein served as the internal control for normalization. The normalized protein level of Neg-ctrl transfectant was set as 1.0 for comparison to those of isomiR-31 transfectants. The data represent the average of 3 independent experiments with standard deviations (*P<0.05; **P<0.01; ***P<0.001, t-test).

Comparing the reported miR-31 isoform sequences in hES/hEB [23], [32], we noticed that the most abundant isoforms of miR-31 differed from the miRBase annotated sequence. In addition, subtle differences in isomiR-31s distribution were observed in HUVEC cells when cultured under hypoxia and normoxic conditions, resulting in a change of the major isoform of miR-31 (Figure 1 and Figure S1). We then analyzed the isoforms of miR-31 in MCF-7 breast cancer cells, HCT116 colon cancer cells, and LNCaP prostate cancer cells by deep sequencing and compared them with the reported isomiR-31s culled from the supplementary data of Morin *et al.* [23], [32]. As shown in Figure 1, the relative abundance of miR-31 isoforms varied among these cells and the most abundant isoform of miR-31 differed between hES/hEB/LNCaP cells and MCF-7/HCT116 cells. We focused on three isomiR-31s in human cells, annotated in miRBase (version 18.0) as the major miR-31 in 3 species, hsa-miR-31, ptr-miR-31, and mmu-miR-31 and dubbed them miR-31-H, miR-31-P and miR-31-M, respectively. Although these three isomiRs differed only slightly at 5′- and 3′-end sequences (Figure 2A), their preponderance varied among different types of human cells (Figure 1).

### IsomiR-31s Display Concordant and Discordant Regulation of Target Genes

To compare the specificity of isomiR-31s on target regulation, we first examined the effects of transfecting cells with the synthetic oligos of these isomiR-31s on 6 known targets of miR-31 in MDA-MB-231 and MCF-7 breast cancer cells which expressed very little endogenous miR-31 (Figure S2A). These known targets included CEBPα, STK40, and E2F2 which had been shown to be downregulated at mRNA level by miR-31-H in ovarian cancer cells [34] and Frizzled3 (Fzd3) and MMP16 which were repressed at the protein level by miR-31 in MDA-MB-231 breast cancer cells [35]. Analysis of the effects of transfecting MDA-MB-231 (Figure 2B) and MCF-7 (Figure 2C) breast cancer cells with isomiR-31s showed a greater repression of STK40 mRNA expression by miR-31-H and miR-31-M to about 22% and 50% of control for MDA-MB-231 and MCF-7 cells, respectively, than by miR-31-P (to 75% and 95% of control in MDA-MB-231 and MCF-7 cells, respectively). E2F2 was also more downregulated by miR-31-H and miR-31-M than by miR-31-P in MDA-MB-231 cells, but inhibited slightly to similar degree by all 3 isomiR-31s in MCF-7 cells. On the other hand, CEBPα was not significantly (*P*>0.05) downregulated by isomiR-31s in MCF-7 cells, but inhibited to similar degree of control by isomiR-31s in MDA-MB-231 cells (69%, 65% and 75% of control by miR-31-H, -P, and –M, respectively; *P*<0.05). Furthermore, transfection of a cell line expressing endogenous isomiR-31s, HCT116 colon cancer cells, revealed that only miR-31-M, but not –H nor –P could significantly repress these 3 target genes (Figure 2D), implying cell type specific regulation of target genes by isomiR-31s. The protein expression of these 3 known targets was also evaluated in isomiR-31s transfected cells. The results showed that the regulation of these 3 known targets by isomiR-31s at mRNA and protein levels was concordant in most but not all cases in MDA-MB-231 (Figure S3A), MCF-7 (Figure S3B), and HCT116 cells (Figure S3C). We also determined the protein expression of 3 other known targets, Fzd3, MMP16, and MCM2 in MDA-MB-231 cells transfected with isomiR-31s. As shown in Figure 2E-G, miR-31-H and –M, but not miR-31-P significantly repressed the expression of Fzd3, MMP16 and MCM2 [36] and the extent of inhibition by miR-31-H and miR-31-M was similar for most of these targets, except that MCM2 was more repressed by miR-31-H than by miR-31-M. These findings indicated that miRNA isoforms exerted different degree of repression of verified target gene of miR-31, even though they possessed identical seed sequence. It is likely that mechanisms in addition to the base-paring of seed region could affect target genes repression by isomiRs (see Discussion). Besides, the inhibitory effects of miR-31-P on most of the above targets were much less than miR-31-H/−M. Hence, these findings provided evidence that isomiR-31s may share identical targets, but also display discriminative regulatory effects on target genes, which may vary in different type of cells.

### IsomiR-31s Differentially Regulate Dicer Expression

Since isomiR-31s displayed differential regulation of some of the known target genes, we sought for novel target of miR-31 to determine if it is differentially regulated by isomiR-31s. Surveying the prediction websites, PicTar [37] and TargetScan [38], we found Dicer to be one of predicted candidates. Although Dicer was reported to be a target gene of miRNA let-7 and miR-103/107 family, we suspected that the regulation of Dicer could be much more complex than the existing evidence.

**Figure 3:**
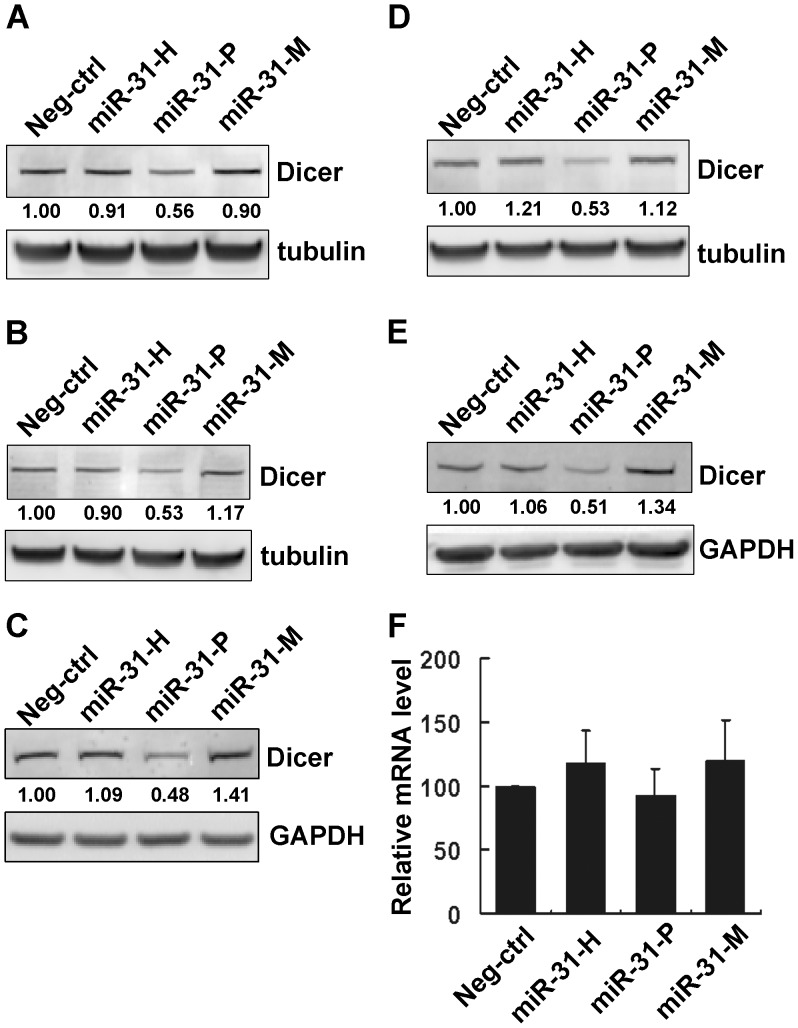
The isomiRs of miR-31 display differential ability in repressing Dicer expression. Immunoblotting of Dicer in MCF-7 cells transfected with 100 nM synthetic oligos purchased from Ambion (A) and Dharmacon (B). Immunoblotting of Dicer in MDA-MB-231 cells (C), in A549 cells (D), and in HCT116 (E) transfected with 100 nM synthetic oligos (Ambion). Relative expression of Dicer mRNA in MCF-7 cells transfected with miRNA synthetic oligos (Ambion) (F). Data were presented as relative expression level to Neg-ctrl transfectant. Neg-ctrl, negative control oligo.

To pursue the possible regulation of Dicer expression by isomiR-31s, MCF-7 cells were transfected with miRNA synthetic oligos, miR-31-H, miR-31-P, or miR-31-M, obtained from the Ambion (see the Materials and Methods) and the expression of Dicer protein was determined. Interestingly, only miR-31-P, but not miR-31-H or miR-31-M, was able to inhibit Dicer expression (Figure 3A). To confirm our finding, oligos of isomiR-31s purchased from another source, Dharmacon, were used in a similar experiment, which confirmed that miR-31-P, but not other 2 isoforms, repressed Dicer expression (Figure 3B). In order to demonstrate that the differential repression effect is not due to unequal transfection efficiency, miR-31 expression level was determined by RT-qPCR, which showed that these three isomiR-31s were indeed equally overexpressed in the transfected cells (Figure S2B and S2C). Similar inhibitory effect of miR-31-P was also observed in MDA-MB-231 breast cancer cells, A549 lung cancer cells, and HCT116 colon cancer cells The latter two cell lines displayed significant level of endogenous miR-31 in contrast to very low level in MCF-7 and MDA-MB-231 (Figure S2A), suggesting that the inhibition of miR-31-P is not restricted to one cell-type nor dependent on endogenous miR-31 level (Figure 3C–E). To ascertain whether miR-31-P repressed Dicer expression at both mRNA and translational levels, we determined the Dicer mRNA expression by RT-qPCR. As shown in Figure 3F, miR-31-P reduced Dicer expression mainly by translational repression, not by mRNA degradation.

**Figure 4:**
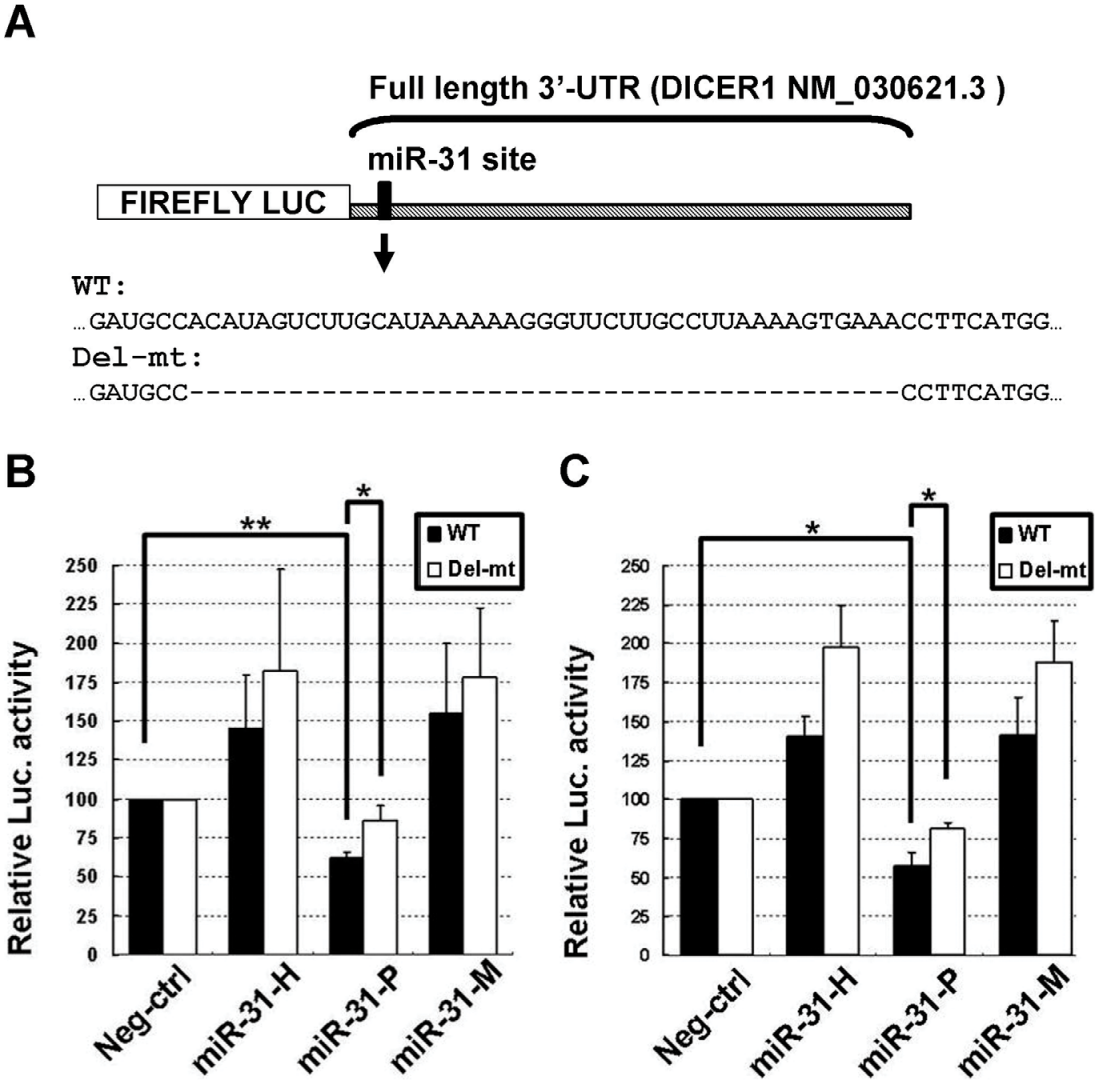
Full length 3′-UTR of Dicer mRNA was differentially repressed by miR-31 isoforms. Full length 3′-UTR of human Dicer mRNA (GenBank accession number NM_030621.3) (Luc-Dicer 3′-WT) is schematically represented. [16] The putative target site (marked by the vertical bar) was predicted by TargetScan. The sequences of full length wild type (WT) and mutant (Del-mt) reporter plasmid were shown in (A). The wild type or mutant reporter plasmid was cotransfected with either miR-31-H, miR-31-P, miR-31-M, or negative control (Neg-ctrl). The normalized luciferase activity of reporter transfected with oligo control (Neg-ctrl) was set to 100%, the reporter activity of other miRNA-transfected groups was relative to it. MiR-31-P repressed the reporter activity of wild type (WT) full length 3′-UTR of Dicer mRNA in MCF-7 (B) and A549 (C) cell lines but not the mutant reporter. The data represent the average of 3 independent experiments with standard deviations (*P<0.05; **P<0.01, t-test).

To further support that isomiR-31s differentially repressed Dicer expression, we performed the reporter assays with full length (∼4,800 nucleotides) 3′-UTR of Dicer mRNA which contains only one miR-31 recognition site, as predicted by TargetScan and PicTar websites. A mutant reporter was constructed by deleting the sequences surrounding the predicted seed region based on RNAhybrid software to ensure complete destruction of the binding for miR-31. As shown in Figure 4, the full length reporter activity was indeed significantly repressed by miR-31-P to 62.3± 3.5% (*P* = 0.004) and 57± 8.6% of control (*P* = 0.019) in MCF-7 (Figure 4B) and A549 (Figure 4C) cell lines, respectively. On the other hand, miR-31-P only slightly reduced the mutant reporter activity to 86.5±9.6% and 80.9±4.0% of control in MCF-7 cells and A549 cells, respectively. These findings indicated that the predicted target site was a genuine target of miR-31. Of note, miR-31-H and miR-31-M appear to promote the luciferase activities of both wild type and mutant reporters, but they had no significant effects on Dicer expression, at either mRNA or protein levels (Figure 3). These findings further strengthened the notion that isomiR-31s can differentially regulate Dicer expression.

### MiR-31-P but not miR-31-H or miR-31-M Enhances Sensitivity of Cancer Cells to Cisplatin

**Figure 5:**
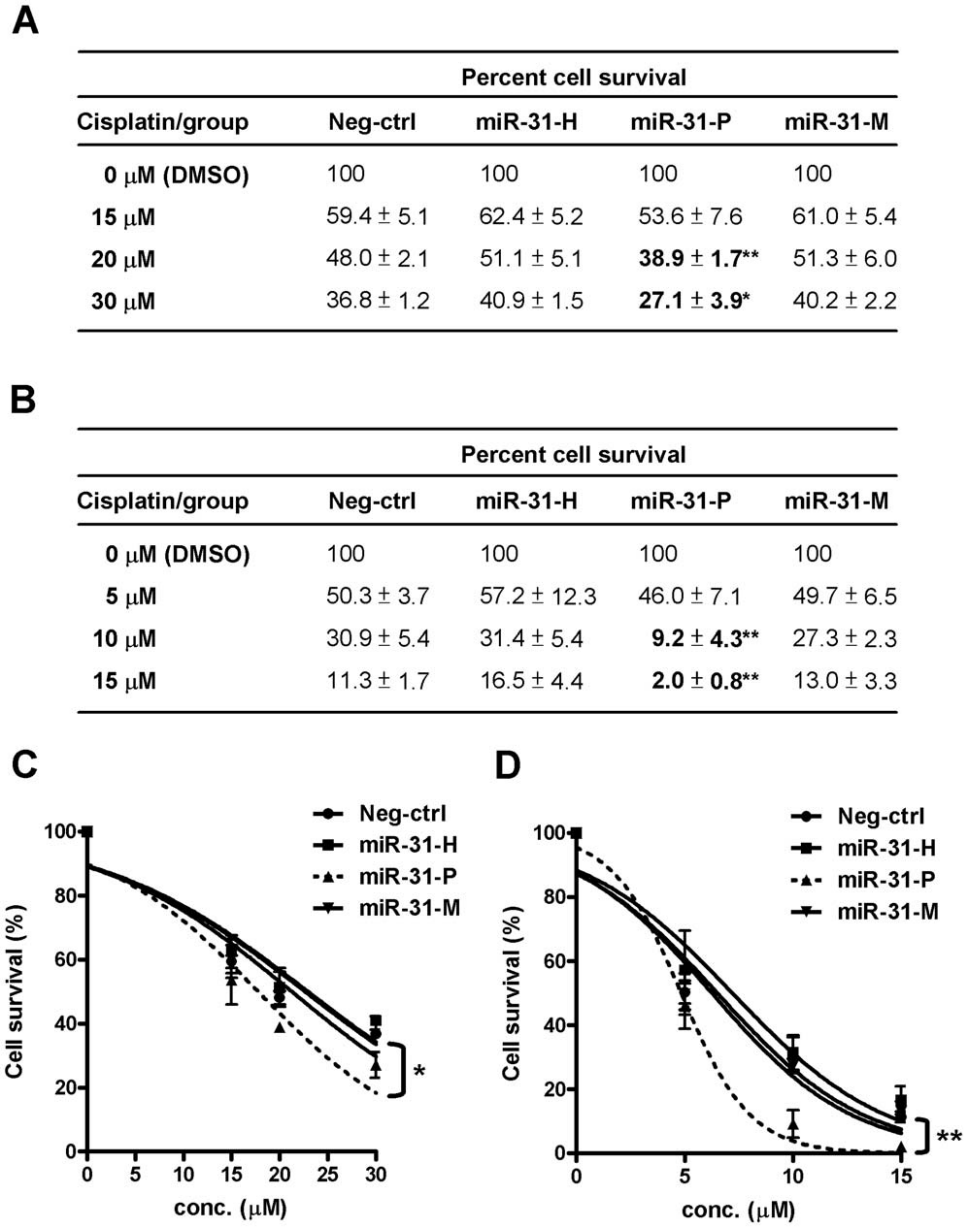
MiR-31-P enhanced the sensitivity of cancer cells to cisplatin treatment. IsomiR-31 transfected MCF-7 breast cancer cells (A) and A549 lung cancer cells (B) were incubated with cisplatin at the indicated concentrations. At 48 h, the numbers of surviving cells were analyzed by Alamar Blue reagent and the percentages of cell survival were listed. The percentage of surviving cells of each transfected groups treated with DMSO was set as 100% to calculate the percentages of surviving cells of cisplatin treated cells at the indicated concentration. Comparing to the negative control transfected cells, miR-31-P enhanced the sensitivity of both cancer cells to cisplatin treatment (*P<0.05; **P<0.01, t-test). The statistical significance of the differential sensitivity to cisplatin of MCF-7 (C) and A549 (D) cells transfected with various isomiR-31s was further examined by nonlinear regression analysis (GraphPad Prism software version 5.01). Nonlinear regression analysis was used to provide the best fitted sigmoid curves by plotting the percentages of cell survival against the drug concentrations (*P<0.05; **P<0.01, ANOVA). The data represent the average of 3 independent experiments with standard deviations.

To explore the biological consequence of negative regulation of Dicer by miR-31-P, we evaluated the possible impact of miR-31-P transfection on the chemosensitivity of cancer cells, in view of the report that Dicer knockdown by siRNA in MCF-7 cells enhanced their sensitivity to cisplatin [39]. After transfection with different isomiR-31s, the sensitivity of MCF-7 cells to cisplatin was assessed. As shown in Figure 5A, the sensitivity of miR-31-H and –M transfected cells to cisplatin was similar to that of cells transfected with control oligo, but the miR-31-P transfected cells were more sensitive in a dose-dependent manner (*P*<0.01 at 20 µM; *P*<0.05 at 30 µM). Using nonlinear regression analysis to provide the best fitted sigmoid curves, plotting the percentages of cell survival against the drug concentrations (Figure 5C) we further confirmed greater cisplatin sensitivity of miR-31-P transfected cells than others (*P*<0.05). Such phenomenon is not restricted to a specific cell type, since miR-31-P transfection also significantly enhanced the sensitivity of A549 lung cancer cell line to cisplatin treatment (*P*<0.01 at both 10 µM and 15 µM; Figure 5B), which was supported by nonlinear regression analysis (*P*<0.01; Figure 5D). These findings suggest that down-regulation of Dicer by miR-31-P contributed at least in part to the increased drug sensitivity.

### The Binding Capacity of isomiR to AGO Complex Might not be the Only Critical Element for the Target Gene Repression

Previous studies indicated that some miRNA variants were differentially loaded onto AGOs and 5′-end nucleotide of small RNA was critical for its interaction with AGO proteins [40]–[42], suggesting the possibility that isomiR-31s which differed in their target gene predilection may display differential binding capacity for AGO complex, which is crucial for target repression. To determine the binding capacity of miR-31 isoform with AGO complex, MCF-7 cells were cotransfected with the plasmid expressing Argonaute2 (AGO2), one of the 3 isomiR-31s oligos, and miR-132 oligo, which served as an internal control for normalization since miR-132 was not a predicted regulator of Dicer and failed to repress Dicer expression by western blot analysis (Figure S4A). The binding of isomiR-31s and miR-132 to AGO2 complexes was determined by RNA-CHIP assay and RT-qPCR. The result of western blot analysis shown in Figure S4B documented efficient transfection and immunoprecipitation of AGO2. Comparing the Ct values of bound isomiR-31s and miR-132 within control-IP samples to those within AGO2-IP samples, the amounts of miRNAs captured within AGO2-IPs were hundred folds higher than those in control-IPs (Ct values ranged from 26.58 to 29.88, and 18.72 to 21.00 for control-IP and AGO2-IP, respectively), indicating that miRNAs indeed were effectively bound within functional AGO2 complexes rather than control vector. After normalization to the internal control miR-132, the percentage of bound miR-31-P was set as 100%. As shown in Figure S4C,the bound miR-31-H within AGO2 complexes was significantly lower than bound miR-31-P (38±9%) (*P*<0.05), whereas, the bound miR-31-M was not significantly different from bound miR-31-P (85±19%) (*P*  =  0.36), suggesting that the 5′-end nucleotide of isomiRs was not an absolute criterion for AGO complex loading (see Discussion).

To eliminate the possibility of differential amplification efficiency of commercially available miR-31-H RT-qPCR probe for the 3 isomiR-31s, we used synthetic single stranded RNAs (ssRNAs) with sequences identical to –H, -P, and –M form (ss-H, ss-P, and ss-M), to mimic the in vitro RT-qPCR analysis. The amplification efficiency of RT-qPCR probe for each ssRNA form was determined with serial dilutions of ssRNA inputs. As shown in Figure S4D, the slope of these 3 qPCR amplification curves were almost identical (3.75 for ss-H; 3.73 for ss-P; 3.85 for ss-M), indicating that the amplification efficiency of the miR-31-H RT-qPCR probe was equally effective for detecting all 3 isomiR-31s. In other words, the higher amount of miR-31-P and -M detected in RNA-CHIP assay was indeed contributed by their higher binding capacity for the AGO complex. Thus, the differential binding capacity of isomiR-31s with Argonaute (AGO) complex was one of, but not a crucial element accounting for the disparate functions of isomiRs.

## Discussion

MiRNAs have emerged as one of the key regulators for gene expression. Before isomiRs were discovered, the miRNA variants were usually missed or ignored by traditional miRNA cloning technique. With the advances in deep sequencing, increasing numbers of miRNAs and its cognate miRNAs, miRNA-3p, were found to differ from the currently annotated sequence in miRBase, and the population of miRNA isoforms varied among different tissues or cell types [23], [43]. However, the possibility of concordant or discordant regulation of target genes by different isoforms of miRNAs has not been validated at the cellular level until this report. In this study, we used miR-31 as a model to demonstrate that the most abundant isoform of miR-31 and its cognate miRNA, miR-31-3p, varied in different cells by comparing our deep sequencing data in MCF-7, HCT116, and LNCaP with the previous report (hES and hEB) (Figure 1 and Figure S5). We further investigated the functions of isomiRs at the cellular level and provided direct evidences that isomiRs are not equal in their target regulation. Previously, it was reported that hundreds of mRNA enriched in the miRNA pull-down were common to their isomiR pull-downs by microarray analysis [33]. However, a close scrutiny of their data revealed that some mRNA targets were unique to the specific isomiRs. Such systems analysis, although powerful, did not offer direct proof for the regulation of a particular target by specific isomiRs. Herein, our studies have provided solid evidence for the complexity of target regulation by isomiRs at the cellular level.

Several inherent challenges in the investigation of isomiRs were encountered in our study. First, traditional cloning and sequencing is not ideal for quantifying isomiRs because cloning frequencies may not truly reflect the isomiR populations. Another technical limitation of traditional cloning is to accurately delineate 5′- or 3′-end sequence information of a specific miRNA (see Figure S6). The use of northern blot analysis is not practical for isomiR study either, since there are no available commercial probes including LNA detection probe that can guarantee specific distinction of our three isomiR-31s. Even if miR-31-M and miR-31-H/−P were distinguishable by northern blotting, it is not possible to separate miR-31-H and miR-31-P from each other because of their identical length. Although the TaqMan qPCR probes were widely used in miRNA studies, we found that the same probe for miR-31-H could also recognize the other two isoforms (Figure S4D). Thus, the specificity of the TaqMan probe is not sensitive enough for our experiments. Hence, deep sequencing is the only reliable approach to identify the endogenous isomiRs populations in different cells or tissue. The second challenge is the limited choice of strategies for overexpressing and silencing specific isomiRs. Since isomiRs were processed from the same pri-miRNA/pre-miRNA, it will not be straightforward to identify specific isomiR-31 generated by transfecting cells with a plasmid bearing pri-miR-31/pre-miR31 sequence, making it difficult to attribute the observed phenotype to any specific isoform after transfection. Instead, we used synthetic double stranded miRNA oligos pledged by Ambion and Dharmacon for transfection into cancer cells to compare the functions of isomiRs. To further confirming our finding by silencing a specific isoform of miR-31 is not feasible either, because of a lack of molecules that are guaranteed to inhibit specific endogenous miRNA isoform. Thus, to address the functions of isomiRs in depth, it may be necessary to simultaneously decipher the expression profile of target genes and the populations of isomiRs in different types of cells, which awaits future studies.

Since gene regulation mediated by miRNA requires the ternary interactions among miRNA/AGO/target mRNA, it is possible that differential interactions of isomiRs within the ternary complex may lead to disparate regulation of target genes. In this study, the observed discrepancy between the miR-31 isoforms bound within AGO-IP and their repression of Dicer and other known target genes suggested that the affinity of a given miRNA to AGO or their seed sequences might not be the only critical elements for the target gene repression. In fact, several factors have been shown to dictate the recognition of target site by miRNA, such as (1) the sequence composition of the 3′-UTR [44], (2) the immediate environment of the putative target site [45], (3) the structural accessibility of the target site [46], [47], and so forth. Besides, endogenous natural antisense transcript (NAT), which was transcribed from the opposite strand of protein-coding gene or non-protein coding gene [48], and the RNA binding proteins [49] could directly bind to mRNA, thereby masking the miRNA binding site of target gene and preventing the inhibitory effects of the miRNA on target gene translation. Although the bindings of miR-31-P and –M to AGO complexes were comparable, the above-mentioned factors might come into play in the differential regulation of Dicer and other known target genes expression. The exact mechanisms underlying the target specificity of isomiRs await further investigation in the future. Taken together, the variations in the relative abundance of isomiRs among different cell types coupled with our finding that isomiRs could differentially regulate the expression of target genes, suggest that isomiRs may play a more general and weighty role in nature by fine-tuning target gene expression.

## Materials and Methods

### Cell Cultures

*MCF-7* breast cancer cell line was cultured in Modified Eagle Medium supplemented with 10% fetal bovine serum, 10 mg/ml insulin, 1% Glutamax, and 1% sodium pyruvate. A549 lung cancer cell line was cultured in RPMI1640 medium supplemented with 10% fetal bovine serum. MDA-MB-231 breast cancer cell line was cultured in Dulbecco’s Modified Eagle Medium supplemented with 10% fetal bovine serum. All cell lines were obtained from American Type Culture Collection (ATCC, Manassas, VA).

### MiRNA Oligos

For isomiR study, miRNA synthetic oligos were purchased from Ambion (Austin, TX, USA) and Dharmacon (Lafayette, CO, USA). All miRNA synthetic oligos from both sources were double strand form and were guaranteed products by manufacturers. The miRNA synthetic oligos of hsa-miR-31 were purchased from Ambion (hsa-miR-31:Cat. #PM11465, ptr-miR-31: Cat. #PM10757, and mmu-miR-31: Cat. #PM10653) and Dharmacon (hsa-miR-31: Cat. #C-300507-05, ptr-miR-31:Cat. #C-120371-00, and mmu-miR-31: Cat. #C-310524-05), and designated as miR-31-H, miR-31-P, and miR-31-M, respectively. For RNA-CHIP assay, pre-miR-132 (Ambion, Cat. #PM10166) was used as an internal control (see the section below).

### Plasmids and Luciferase Reporter Assay

The flag-AGO2 plasmid was kindly provided by Dr. S. C. Lu (National Taiwan University, Taiwan). The full length 3′-UTR of wild type Dicer reporter plasmid (Luc-Dicer 3′-WT) was a generous gift from Dr. Piccolo [16]. The mutant 3′-UTR reporter was generated by using the QuickChange XL Site-Directed Mutagenesis Kit (Stratagene, La Jolla, CA, USA) with primer pairs, Del-mt F/Del-mt R, according to the manufacturer’s instruction. All primer sequences are listed in Table S1. For reporter assay, 0.2 µg of Luc-Dicer 3′-WT or 0.2 µg of mutant reporter plasmid (Del-mt) was cotransfected with 0.2 µg of phRG-TK vector (internal control for normalization) and miRNA oligos (20 nM final concentration) (Ambion) by lipofectamine 2000 (Invitrogen, Carlsbad, CA, USA). Forty-eight hours after transfection, cells were harvested and the luciferase activity was determined by Dual-Luciferase Reporter Assay System (Promega).

### Western Blot Analysis

The western blot analysis was conducted as described previously [12], [50]. Forty microgram of cell lysate of each sample was separated by 4–12% *gradient NuPAGE* (Invitrogen). To detect Dicer and internal control tubulin proteins, the primary antibody to Dicer was purchased from Abcam Inc. (ab14601; Abcam, Cambidge, MA, USA), and the antibody to tubulin was purchased from Sigma (clone B-5-1-2; Sigma, St Louis, MO, USA). The antibodies for Fzd3 protein and MMP16 protein were purchased from GeneTex Inc. (GTX100182 and GTX109378, respectively; GeneTex, San Antonio, TX, USA). The antibodies for detecting MCM2 protein and the internal control GAPDH protein were purchased from Epitomics Inc. (2901-1 and 2251-1, respectively; Epitomics, Burlingame, CA, USA). The signal of protein bands was revealed by ECF western blotting kit (Amersham Biosciences, Piscataway, NJ, USA) and measured by Typhoon 9400 imager (Amersham Biosciences).

### RNA-CHIP Assay

Ten microgram of flag-CMV2 or flag-AGO2 plasmid was cotransfected with 80 nM isomiR-31 oligos (-H, -P, or –M, individually) as well as 20 nM miR-132 oligos (as an internal control) into MCF-7 cells. Transfected cells were harvested 72 h after transfection. Before cells lysis, ∼10^5^ cells were collected for RNA extraction by Trizol (Invitrogen) and designated as “RNA-input part”. The remaining cells were treated with lysis buffer (150 mM KCl, 25 mM Tris-HCl (pH 7.4), 5 mM EDTA, 0.5% NP-40, 5 mM DTT, and 1×protease inhibitor) for 30 min and the cell lysates were separated by centrifugation at 12,000 g for 20 min at 4°C. Forty microgram of cell lysate was collected as “PROTEIN-input part” for the following western blot analysis with the flag-specific antibody (F3165; Sigma) to confirm the expression of transfected AGO2 plasmid. Twenty-five microliter of Protein G Dynabeads (Invitrogen) and 4 µg of flag–specific antibody were added to 1 mg cell lysate (in a final 1 ml mixture filled with lysis buffer) and the mixture was rotated for overnight at 4°C. The beads were washed three times with 1 ml lysis buffer to remove non-specific binding. After washings, the beads were resuspended in 1 ml lysis buffer and 50 µl (5% of total volume) of the suspension were collected as “PROTEIN-IP part” for western blot analysis with the flag-specific antibody (F3165; Sigma) to confirm the efficiency of AGO2 immunoprecipitation in each sample. The RNAs bound on the remaining beads were extracted by Trizol and the RNAs were precipitated with linear acrylamide (Ambion), which was designated as “RNA-IP part”. MiRNA expression of both INPUT and IP part RNAs were analyzed by RT-qPCR (as described below). AGO2 proteins of both INPUT and IP parts were analyzed by the western blot analysis.

### RT-qPCR Assay and Analysis

Ten nanogram of total RNA was used for quantification of miRNAs expression, including isomiR-31s, miR-132 and RNU6B (U6) RNA, by TaqMan RT-qPCR kit (Assay ID 002279 for all miR-31 isoforms, Assay ID 000457 for miR-132, and Assay ID 001093 for RNU6B; Applied Biosystems, Foster City, CA, USA) according to the manufacturer’s instruction. For RNA-CHIP assay, the normalized miR-31 amount in each IP was calculated as the ΔCt ( = Ct_miR31_– Ct_miR132_). The amount of bound miR-31-P in the AGO2/miR-31-P cotransfectant was set as 1.0 and the relative amount of bound isomiR-31 in AGO2/miR-31-H or AGO2/miR-31-M cotransfectants was calculated by the formula: 2^−(ΔCt of AGO2/miR31H or M − ΔCt of AGO2/miR31P)^. For the quantitation of mRNAs of Dicer, CEBPα, STK40, and E2F2, 1 µg of total RNAs were reverse transcribed into cDNA using the SuperScript III kit (Invitrogen) and the specific mRNAs were detected by Power SYBR Green PCR Master Mix (Applied Biosystems) according to the manufacturer’s instruction. The primer sequences and PCR condition of Dicer qPCR were performed as described previously [9]. The primers for CEBPα, STK40, and E2F2 detections were as designed on the OriGene website (http://www.origene.com/). The RT-qPCR was performed on the 7300 Real-Time PCR System (Applied Biosystems).

### MiRNA Deep Sequencing

MiRNA was isolated from the total RNA sample using mirVana™ miRNA Isolation Kit (Ambion, AM1561) and subsequently constructed into fragment sequencing library using the procedure of SOLiD™ Small RNA Expression Kit (Applied Biosystems, 4397682). Procedure for fragment sequencing library construction, including template bead preparation, emulsion PCR, bead deposition and sequencing by SOLiD^TM^3 system (Applied Biosystems), was based on the standard protocol provided by the company.

### Cisplatin Resistance Determination and Alamar Blue Assay

Cells were transfected with 100 nM synthetic isomiR-31 oligos (Ambion). Seventy-two hours after transfection, cells were incubated with indicated concentrations of cisplatin (Sigma) for 48 h. Cell viability was analyzed by Alamar Blue reagent (Biosource International, Camarillo, CA, USA) according to the manufacturer’s instruction. The percentage of surviving cells of each transfected groups treated with DMSO was set as 100% to calculate the percentages of surviving cells of cisplatin treated groups at the indicated concentration by the following formula: (the OD_590_ value in drug group/the OD value in DMSO solvent control group)×100%, respectively. To further assess the statistical significance of differential cisplatin sensitivity of cells transfected with various isomiR-31s, the nonlinear regression model and the classic equation of “sigmoid dose-response (variable slope)” were chosen, and then the sigmoid concentration response curves were generated using GraphPad Prism software version 5.01 (GraphPad, La Jolla, CA, USA). Before fitting the dose-response curves, the parameter of logEC50 was selected for asking the significant difference among each data set. Moreover, the top and bottom of best-fit values were constrained as 100 and 0 for fitting the top and bottom plateau of the curves. The statistical significance of best fitted curves between miR-31-P transfected cells and other groups was determined.

## Supporting Information

Figure S1 **The sequences of most abundant isoforms of miRNAs differ among various cells and type of culture conditions within the same cell.** (A) Based on the report of Azuma-Mukai *et al.*, the sequence of the most abundant form of miR-142-5p in Jurkat cells differs from the miRBase annotation (version 18.0) in 5′- and 3′-end [22], [30]. Our deep sequencing data in MCF-7, HCT116 and LNCaP cell lines showed that the most abundant forms of miR-31 in hES/hEB/LNCaP are different from that in MCF-7/HCT116. The latter is identical to the miRBase annotated sequence (version 18.0) [23], [30]. The most abundant isoforms of miR-151-5p differ in Jurkat and hES/hEB cells and both of which differ from miRBase annotation [22], [23], [30]. (B) The most abundant isoform of miR-30b-5p, miR-455-3p, and miR-31 in HUVEC cells differs under hypoxia and normal culture condition [32]. The isoforms sequence mismatching to precursor sequence due to SNPs or editing-events were excluded from this table.
(TIF)

Figure S2 **The expression levels of miR-31 as detected by RT-qPCR.** The expression levels of endogenous miR-31 in MCF-7, MDA-MB-231, A549, and HCT116 cancer cells (A). The level of overexpressed isomiR-31s in MCF-7 cells transfected with synthetic oligos from Ambion (B) and Dharmacon (C). The expression level was shown as miR-31 (−ΔCt), which is equal to – (Ct_miR-31_−Ct_U6_).
(TIF)

Figure S3 **The regulation of 3 known targets including CEBPα, STK40, and E2F2 by isomiR-31s at protein levels in MDA-MB-231 (A), MCF-7 (B), and HCT116 (C) cell lines.** GAPDH or tubulin protein served as the internal control for normalization. The normalized protein level of Neg-ctrl transfectant was set as 1.0 for comparison to those of isomiR-31 transfectants.
(TIF)

Figure S4 **IsomiRs have differential binding abilities to the AGO complex.** (A) Immunoblotting of Dicer in MCF-7 cells transfected with Negative control (Neg-ctrl) or miR-132 oligo. Tubulin protein served as the internal control for normalization. (B) The transfection condition was as indicated in the upper panel. The transfection and immunoprecipitation procedures were confirmed by the western blotting. Forty microgram of total cell lysate of each sample before IP procedure was used as the input control and 5% of the IP product was used as the IP control for the following western blot analysis. Flag-AGO2 protein was detected by the flag–specific antibody. (C) The relative amounts of bound miR-31 isoforms in AGO2-IP products. The bound miR-31 isoform was detected by RT-qPCR assay. After normalizing to the miR-132 internal control, the amount of bound miR-31-P was set as 100% and the others were relative to it. The data represent the average of 3 independent experiments with standard deviations (*P<0.05, t-test). (D) The miR-31 RT-qPCR probes for detecting of miR-31 isoforms have similar amplification efficiencies. Synthetic single strand RNAs with sequences corresponding to miR-31-H, miR-31-P, and miR-31-M were denoted as ss-H, ss-P, and ss-M, respectively. X-axis indicated the concentration of single strand RNA input, y-axis indicated the Ct value of RT-qPCR detection. The regression line of qPCR amplification for each ssRNA template was calculated and shown. The amplification efficiency of RT-qPCR probe for each ssRNA form was determined with the serial dilutions of ssRNA inputs and is shown as the regression line. The slope of these 3 qPCR amplification lines were almost identical (3.75 for ss-H; 3.73 for ss-P; 3.85 for ss-M), indicating that the amplification efficiency of this RT-qPCR probe was fairly similar for the detection of these 3 isomiR-31s.
(TIF)

Figure S5 **The differential populations of miR-31-3p isoforms, the cognate miRNA of miR-31, in human cell lines.** The isoforms of miR-31-3p in MCF-7, HCT116, and LNCaP cells were identified by deep sequencing. The isomiR-31-3p information of hES/hEB cells was culled from the supplementary data of Morin *et al.* [23]. The sequence underlined with thick lines or marked with ^&^ is the current annotated miR-31-3p of human in the miRBase (version 18.0). All the most abundant miR-31-3p sequence of hES/hEB and MCF-7/HCT116 cells are not identical to the annotated sequence of the miRBase (version 18.0). The occurrence of each sequence read is represented as the count shown in number. In HCT116 profile, most of sequences, which the counts were less than 10, were omitted from this figure. The percentage of each sequence indicates its occurrence in the whole population of miR-31-3p isoforms.^ #^, the data were culled from the report of Morin *et al.*
(TIF)

Figure S6 **The cloning scheme of isomiR-31s were plotted to show that traditional cloning and sequencing is not ideal for identifying a specific miRNA isoforms.** The converting procedure of miRNAs/small RNAs into detectable cDNA was shown in the upper panel. After the cDNA pool of small RNAs was generated, isomiR-31s could specific tag and amplify by (A) 5′ primer (the primer sequence was complemented to 5′ adaptor and 5′-end of miR-31) and 3′ primer (the sequence was complemented to 3′ poly A adaptor), or by (B) 5′ primer (the sequence was complemented to 5′ adaptor) and 3′ primer (the sequence was complemented to 3′ poly A adaptor and 3′-end of miR-31) from the cDNA library for miR-31 cloning. However, using primer set A or B would loss the 5′-end or 3′-end information of the isomiR-31s, respectively.
(TIF)

Table S1 **Primers sequence.**
(DOC)
