# Supplementary figures and images for: Concordant and Discordant Regulation of Target Genes by miR-31 and Its Isoforms
Source: PLoS One. 2013 Mar 5;8(3):e58169. doi: 10.1371/journal.pone.0058169 (PMC3589381; doi:10.1371/journal.pone.0058169)

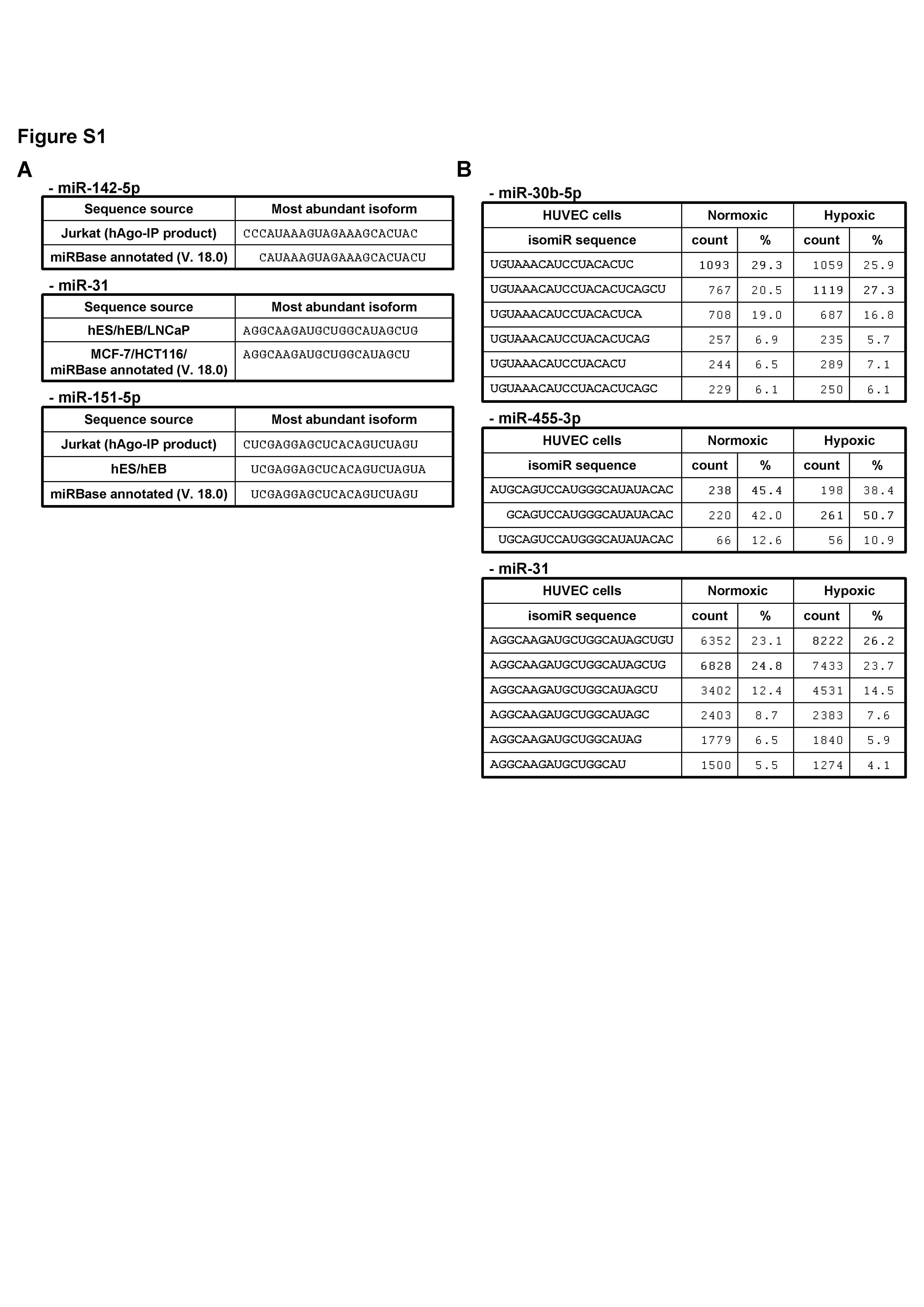

Supplement: Figure S1 — The sequences of most abundant isoforms of miRNAs differ among various cells and type of culture conditions within the same cell. (A) Based on the report of Azuma-Mukai et al., the sequence of the most abundant form of miR-142-5p in Jurkat cells differs from the miRBase annotation (version 18.0) in 5′- and 3′-end [22], [30]. Our deep sequencing data in MCF-7, HCT116 and LNCaP cell lines showed that the most abundant forms of miR-31 in hES/hEB/LNCaP are different from that in MCF-7/HCT116. The latter is identical to the miRBase annotated sequence (version 18.0) [23], [30]. The most abundant isoforms of miR-151-5p differ in Jurkat and hES/hEB cells and both of which differ from miRBase annotation [22], [23], [30]. (B) The most abundant isoform of miR-30b-5p, miR-455-3p, and miR-31 in HUVEC cells differs under hypoxia and normal culture condition [32]. The isoforms sequence mismatching to precursor sequence due to SNPs or editing-events were excluded from this table. (TIF) [file pone.0058169.s001.tif]

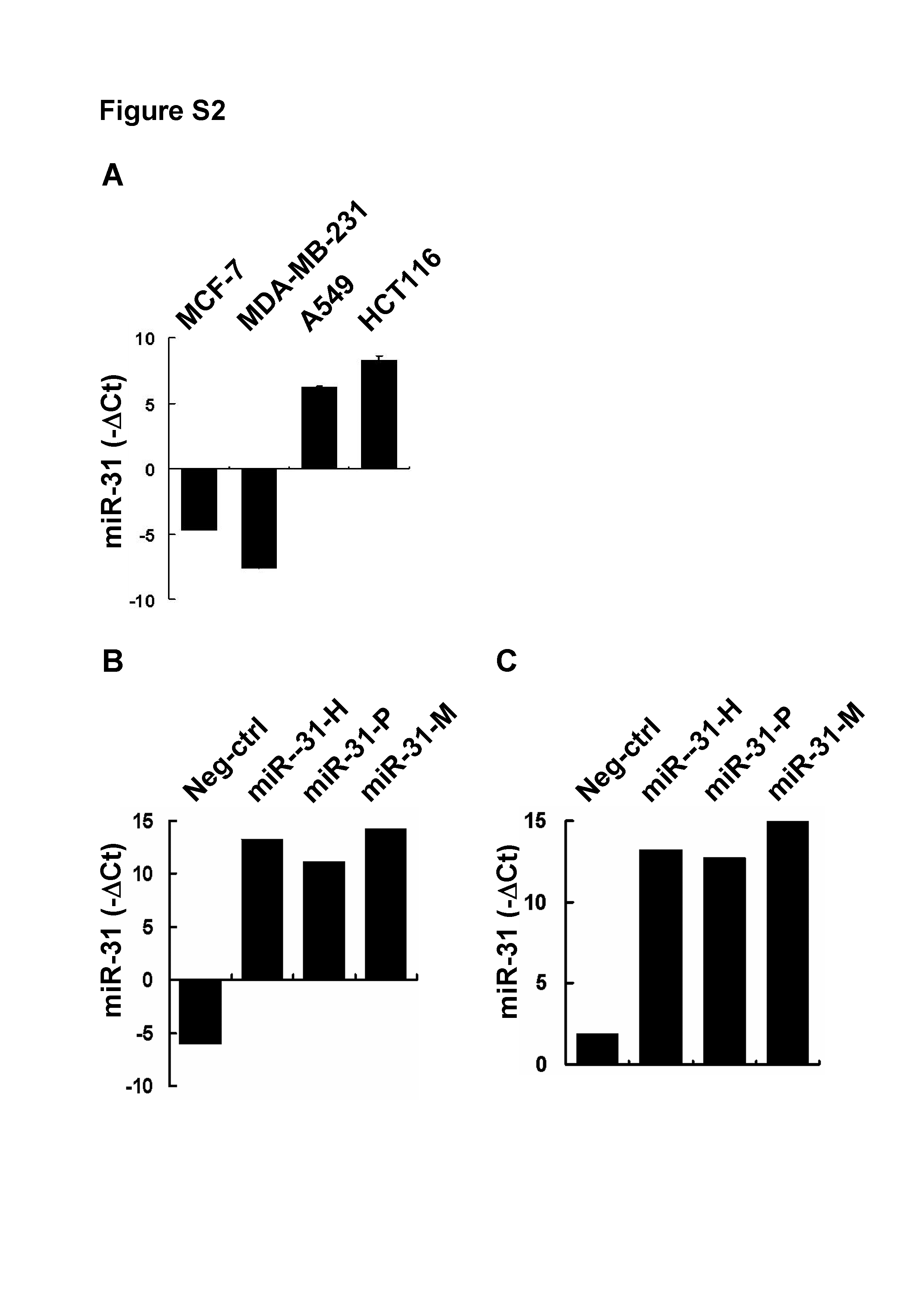

Supplement: Figure S2 — The expression levels of miR-31 as detected by RT-qPCR. The expression levels of endogenous miR-31 in MCF-7, MDA-MB-231, A549, and HCT116 cancer cells (A). The level of overexpressed isomiR-31s in MCF-7 cells transfected with synthetic oligos from Ambion (B) and Dharmacon (C). The expression level was shown as miR-31 (−ΔCt), which is equal to – (CtmiR-31−CtU6). (TIF) [file pone.0058169.s002.tif]

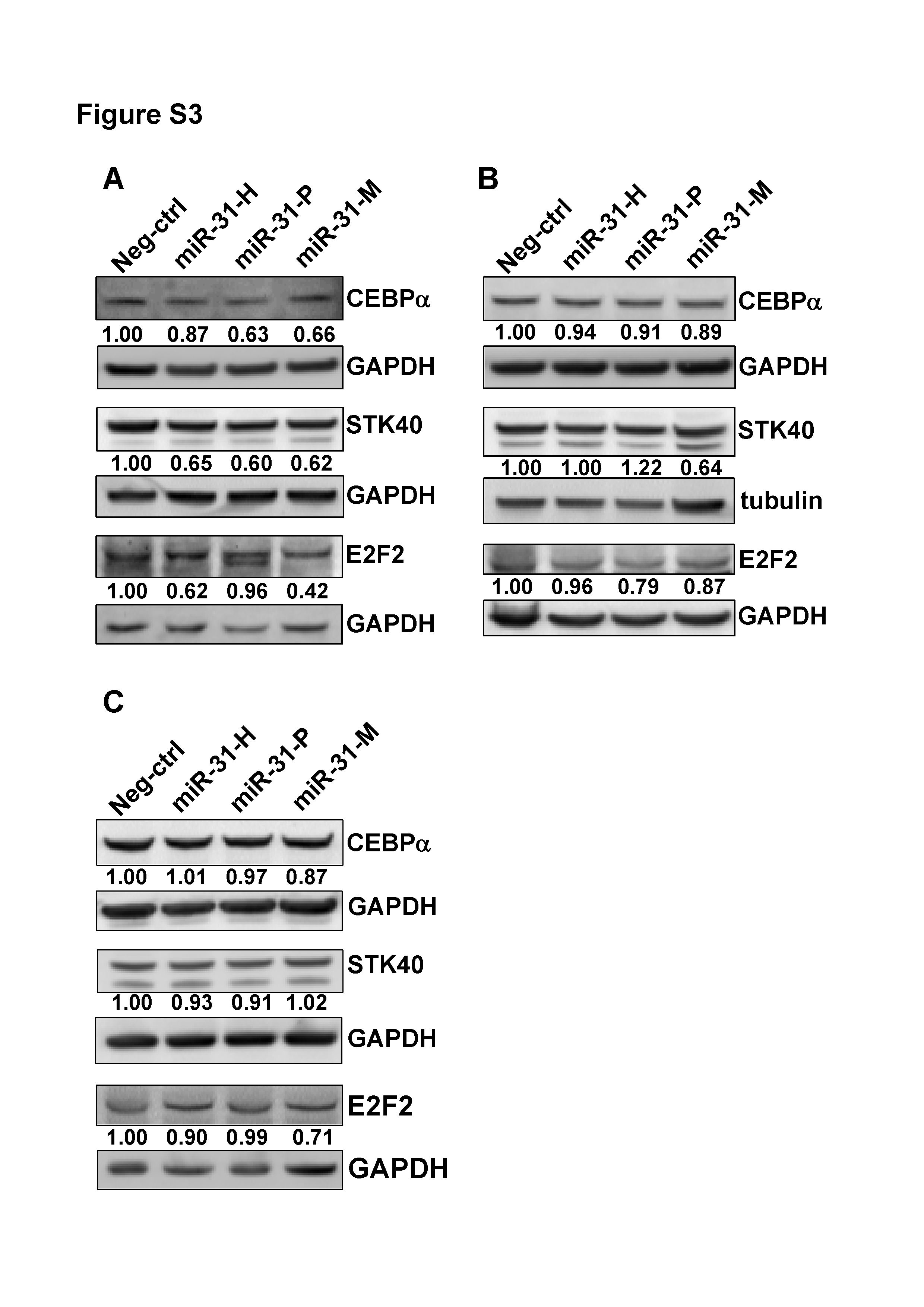

Supplement: Figure S3 — The regulation of 3 known targets including CEBPα, STK40, and E2F2 by isomiR-31s at protein levels in MDA-MB-231 (A), MCF-7 (B), and HCT116 (C) cell lines. GAPDH or tubulin protein served as the internal control for normalization. The normalized protein level of Neg-ctrl transfectant was set as 1.0 for comparison to those of isomiR-31 transfectants. (TIF) [file pone.0058169.s003.tif]

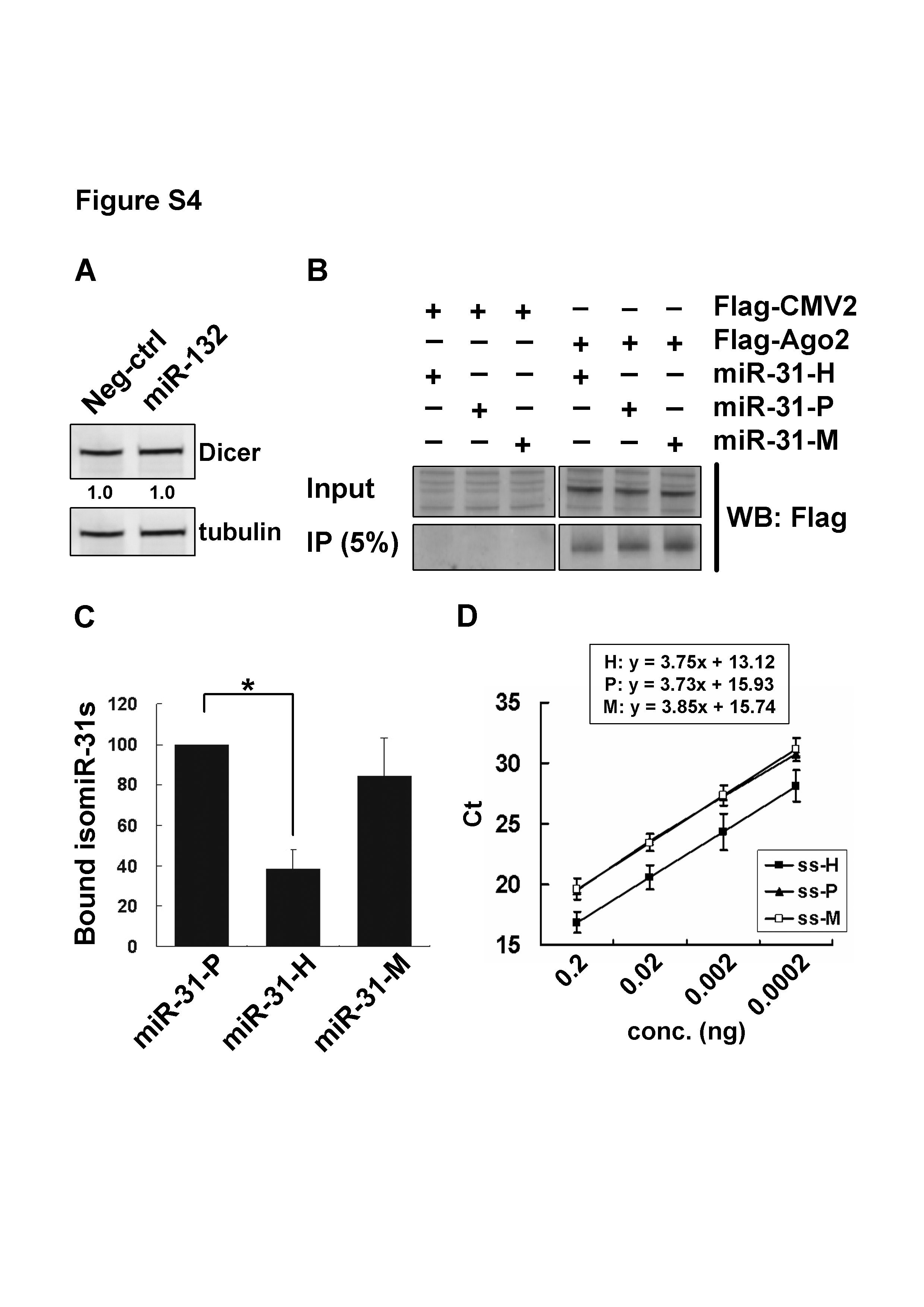

Supplement: Figure S4 — IsomiRs have differential binding abilities to the AGO complex. (A) Immunoblotting of Dicer in MCF-7 cells transfected with Negative control (Neg-ctrl) or miR-132 oligo. Tubulin protein served as the internal control for normalization. (B) The transfection condition was as indicated in the upper panel. The transfection and immunoprecipitation procedures were confirmed by the western blotting. Forty microgram of total cell lysate of each sample before IP procedure was used as the input control and 5% of the IP product was used as the IP control for the following western blot analysis. Flag-AGO2 protein was detected by the flag–specific antibody. (C) The relative amounts of bound miR-31 isoforms in AGO2-IP products. The bound miR-31 isoform was detected by RT-qPCR assay. After normalizing to the miR-132 internal control, the amount of bound miR-31-P was set as 100% and the others were relative to it. The data represent the average of 3 independent experiments with standard deviations (*P<0.05, t-test). (D) The miR-31 RT-qPCR probes for detecting of miR-31 isoforms have similar amplification efficiencies. Synthetic single strand RNAs with sequences corresponding to miR-31-H, miR-31-P, and miR-31-M were denoted as ss-H, ss-P, and ss-M, respectively. X-axis indicated the concentration of single strand RNA input, y-axis indicated the Ct value of RT-qPCR detection. The regression line of qPCR amplification for each ssRNA template was calculated and shown. The amplification efficiency of RT-qPCR probe for each ssRNA form was determined with the serial dilutions of ssRNA inputs and is shown as the regression line. The slope of these 3 qPCR amplification lines were almost identical (3.75 for ss-H; 3.73 for ss-P; 3.85 for ss-M), indicating that the amplification efficiency of this RT-qPCR probe was fairly similar for the detection of these 3 isomiR-31s. (TIF) [file pone.0058169.s004.tif]

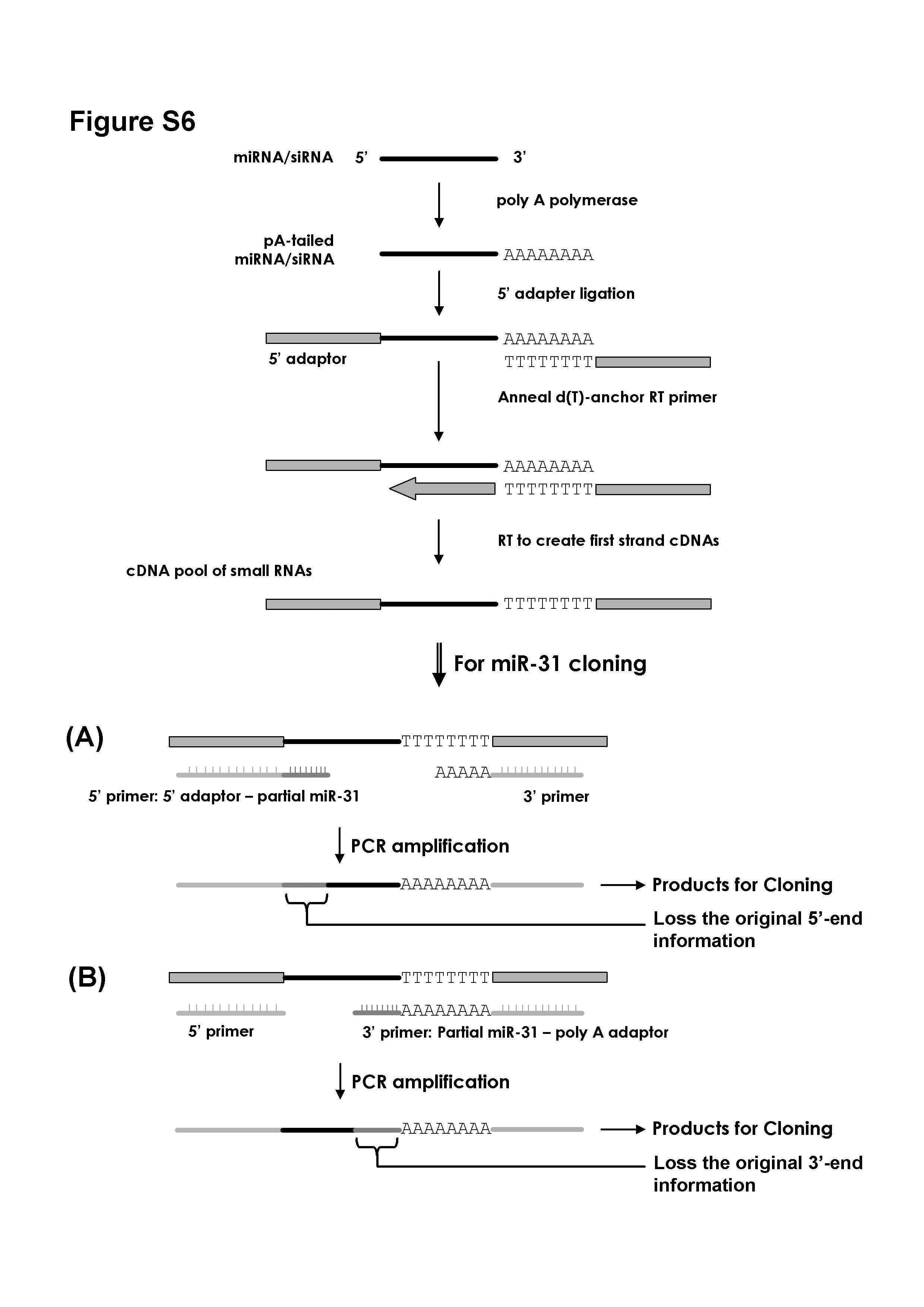

Supplement: Figure S6 — The cloning scheme of isomiR-31s were plotted to show that traditional cloning and sequencing is not ideal for identifying a specific miRNA isoforms. The converting procedure of miRNAs/small RNAs into detectable cDNA was shown in the upper panel. After the cDNA pool of small RNAs was generated, isomiR-31s could specific tag and amplify by (A) 5′ primer (the primer sequence was complemented to 5′ adaptor and 5′-end of miR-31) and 3′ primer (the sequence was complemented to 3′ poly A adaptor), or by (B) 5′ primer (the sequence was complemented to 5′ adaptor) and 3′ primer (the sequence was complemented to 3′ poly A adaptor and 3′-end of miR-31) from the cDNA library for miR-31 cloning. However, using primer set A or B would loss the 5′-end or 3′-end information of the isomiR-31s, respectively. (TIF) [file pone.0058169.s006.tif]
