# Supplementary material for: Concordant and Discordant Regulation of Target Genes by miR-31 and Its Isoforms
Source: PLoS One. 2013 Mar 5;8(3):e58169. doi: 10.1371/journal.pone.0058169 (PMC3589381; doi:10.1371/journal.pone.0058169)
Supplement: Table S1 — Primers sequence. (DOC) [file pone.0058169.s007.doc]

**Table S1**

mutagenesis

AGATTAAAGACTATCCATGAAGGCATCAGAAAACTAAAATGT

**R**

mutagenesis

ACATTTTAGTTTTCTGATGCCTTCATGGATAGTCTTTAATCT

**F**

**Del-mt**

**Sequence (5' to 3')**

**Primer**

**Reporters**

qPCR detection

ACCTTCCCCATGGTGTCTGA

**R**

qPCR detection

CTGCTCCTCCTGTTCGACAGT

**F**

**GAPDH**

qPCR detection

CTTGACGGCAATCACTGTCTGC

**R**

qPCR detection

CTCTCTGAGCTTCAAGCACCTG

**F**

**E2F2**

qPCR detection

TTCCTGGCTCTCTATGCCTTGG

**R**

qPCR detection

GTGTTTGGCGAGGAAAGATGGC

**F**

**STK40**

qPCR detection

AGTGCGCGATCTGGAACTGCAG

**R**

qPCR detection

AGGAGGATGAAGCCAAGCAGCT

**F**

**CEBPα**

qPCR detection

ATAGTACACCTGCCAGACTGT

**R**

qPCR detection

GTACGACTACCACAAGTACTTC

**F**

**Dicer**

**Sequence (5' to 3')**

**Primer**

**qPCR**
